# Human evolutionary loss of epithelial Neu5Gc expression and species-specific susceptibility to cholera

**DOI:** 10.1371/journal.ppat.1007133

**Published:** 2018-06-18

**Authors:** Frederico Alisson-Silva, Janet Z. Liu, Sandra L. Diaz, Lingquan Deng, Mélanie G. Gareau, Ronald Marchelletta, Xi Chen, Victor Nizet, Nissi Varki, Kim E. Barrett, Ajit Varki

**Affiliations:** 1 Glycobiology Research and Training Center (GRTC), Center for Academic Research and Training in Anthropogeny (CARTA), Departments of Medicine and Cellular & Molecular Medicine, University of California San Diego, La Jolla, CA, United States of America; 2 Department of Pediatrics, University of California San Diego, La Jolla, CA, United States of America; 3 Division of Gastroenterology, Department of Medicine, University of California San Diego, La Jolla, CA, United States of America; 4 Department of Chemistry, University of California Davis, Davis CA, United States of America; 5 Skaggs School of Pharmacy and Pharmaceutical Sciences, University of California San Diego, La Jolla, CA, United States of America; Northwestern University, Feinberg School of Medicine, UNITED STATES

## Abstract

While infectious agents have typical host preferences, the noninvasive enteric bacterium *Vibrio cholerae* is remarkable for its ability to survive in many environments, yet cause diarrheal disease (cholera) only in humans. One key *V*. *cholerae* virulence factor is its neuraminidase (VcN), which releases host intestinal epithelial sialic acids as a nutrition source and simultaneously remodels intestinal polysialylated gangliosides into monosialoganglioside GM1. GM1 is the optimal binding target for the B subunit of a second virulence factor, the AB_5_ cholera toxin (Ctx). This coordinated process delivers the CtxA subunit into host epithelia, triggering fluid loss via cAMP-mediated activation of anion secretion and inhibition of electroneutral NaCl absorption. We hypothesized that human-specific and human-universal evolutionary loss of the sialic acid *N*-glycolylneuraminic acid (Neu5Gc) and the consequent excess of *N*-acetylneuraminic acid (Neu5Ac) contributes to specificity at one or more steps in pathogenesis. Indeed, VcN was less efficient in releasing Neu5Gc than Neu5Ac. We show enhanced binding of Ctx to sections of small intestine and isolated polysialogangliosides from human-like Neu5Gc-deficient *Cmah*^*-/-*^ mice compared to wild-type, suggesting that Neu5Gc impeded generation of the GM1 target. Human epithelial cells artificially expressing Neu5Gc were also less susceptible to Ctx binding and CtxA intoxication following VcN treatment. Finally, we found increased fluid secretion into loops of *Cmah*^*-/-*^ mouse small intestine injected with Ctx, indicating an additional direct effect on ion transport. Thus, *V*. *cholerae* evolved into a human-specific pathogen partly by adapting to the human evolutionary loss of Neu5Gc, optimizing multiple steps in cholera pathogenesis.

## Author summary

Ingestion of food or water contaminated with the bacterium *Vibrio cholerae* can cause fatal diarrheal disease (cholera) in humans, but not in other mammals exposed to the bacterium. This unusual pathogen uses a specialized enzyme *V*. *cholerae* neuraminidase (VcN) to cleave sugars called sialic acids and thereby remodel host intestinal glycans, generating high levels of ganglioside GM1, the optimal ligand for the second secreted molecule (cholera toxin, Ctx) that causes diarrhea. Humans lost the ability to produce a kind of sialic acid called Neu5Gc during our evolution and the glycans of our small intestine are different from most other mammals. We hypothesized that *V*. *cholerae* evolved into a human-specific pathogen by adapting its virulence factor to optimally target human intestinal glycans. We found that VcN remodeling of gangliosides into the Ctx ligand was slowed by the presence of Neu5Gc. In keeping with this finding, incorporation of Neu5Gc into human cells impedes facilitation of Ctx intoxication by VcN. Finally, human-like Neu5Gc–deficient *Cmah*^*-/-*^ mouse intestine was more susceptible to Ctx-induced intoxication, implicating a second, as yet undefined, mechanism dependent on the Neu5Gc loss in humans. Thus, the unique glycan composition of human cells and human-like Neu5Gc-deficient mice influences many steps of cholera pathogenesis. This could help explain, at least in part, Robert Koch’s failure to find any nonhuman animal model susceptible to diarrhea caused by *V*. *cholerae*.

## Introduction

Cholera is a life-threatening, human-specific disease caused by the noninvasive enteric bacterium *Vibrio cholerae* that affects millions of people worldwide [1]. Humans are infected after ingestion of food or water contaminated with the pathogen. Bacteria that survive passage through the acidic milieu of the stomach can colonize and multiply on the surface of the small intestinal epithelium [2] and induce severe watery diarrhea. This main symptom of the disease leads to loss of electrolytes and blood volume depletion, and can be fatal if the individual is not rehydrated rapidly [2]. Cholera diarrhea is triggered after the B subunits of cholera toxin (Ctx) bind to the monosialoganglioside, GM1 [3–5], expressed on the outer leaflet of the apical membrane of intestinal epithelial cells, which facilitates toxin endocytosis and retrograde phagosomal transport to the endoplasmic reticulum (ER) [6, 7]. In the ER, the enzymatically active A subunit of Ctx (CtxA) is released from the B subunits and translocated into the cytoplasm, where it activates adenylate cyclase, leading to increased levels of cyclic adenosine monophosphate (cAMP). The rise in cAMP causes intense secretion of chloride ions through the cystic fibrosis transmembrane conductance regulator (CFTR) as well as inhibition of electroneutral sodium chloride absorption [8]. These events are followed by passive water flow in response to osmotic gradients, resulting in profuse diarrhea [9]. CtxA further induces epithelial cell barrier disruption by inhibiting exocyst-mediated trafficking of host proteins that make up the intercellular junctions of epithelial cells, a mechanism that may act in parallel with Cl^−^ secretion to drive the pathophysiology of cholera [10].

The pathogenesis of cholera-induced diarrhea is initiated when Ctx binds to GM1. However, this mono-sialoganglioside is naturally expressed only at low levels in the small intestine compared to di- and tri-sialogangliosides such as GD1a, GD1b, GT1b, etc. [11]. Pioneering studies on the tissue ligand for Ctx demonstrated that *V*. *cholerae* uses its uniquely evolved neuraminidase (VcN) to hydrolyze α2-3- and α2-8-linked sialic acids from complex gangliosides, thereby generating high concentrations of GM1 and favoring the binding and internalization of Ctx by mammalian cells [4, 12]. Thus, VcN-mediated cleavage of sialic acids from multisialylated gangliosides (selectively sparing the one internal α2-3-linked sialic acid that defines GM1) acts synergistically with Ctx in the induction of diarrhea.

Sialylated glycans (such as on glycoproteins and glycosphingolipids) from human cells terminate with only the *N*-acetylneuraminic acid (Neu5Ac) isoform of sialic acid, a pattern that differs from the majority of other mammals, whose glycans can also terminate with *N*-glycolylneuraminic acid (Neu5Gc, which differs from Neu5Ac by one additional oxygen atom) [13]. Humans do not naturally express glycoconjugates containing Neu5Gc because they lack the enzyme CMP-*N*-acetylneuraminic acid hydroxylase (CMAH) due to an Alu-mediated exon deletion in the *CMAH* gene [14]. Thus, while gangliosides from other adult mammals such as calf, pigs and rabbits can terminate in α2-3- and α2-8-linked Neu5Gc or Neu5Ac [15, 16], only α2-3- and α2-8-linked Neu5Ac are found in human gangliosides [17].

Cholera is a human-specific disease. In fact, Robert Koch, one of the founders of modern microbiology, famously wrote in 1884: *“…although these experiments were constantly repeated with material from fresh cholera cases*, *our mice remained healthy*. *We then made experiments on monkeys*, *cats*, *poultry*, *dogs and various other animals […] but we were never able to arrive at anything in animals similar to the cholera process”* [18]. Experimental high dose (~1 x 10^9^ organisms) oral infection of cimetidine-treated 3-day-old infant rabbits with *V*. *cholerae* can lead to lethal watery diarrhea [19]. However, to the best of our knowledge no reports of cholera diarrhea in other adult mammals have been available, nor any theories to explain the human specificity for this disease, which remains a long-standing mystery––a rare failure of Koch to fulfill his own “postulates” for defining an infectious disease agent.

Previous studies from our group have shown that polymers of α2-8-linked Neu5Gc are much less susceptible to cleavage by mammalian and bacterial neuraminidases compared to polymers containing α2-8-linked Neu5Ac [20]. These findings led us to hypothesize that the difference in the sialic acid composition between the glycans of humans and other mammals influences the ability of VcN to generate epitopes for Ctx binding. This, together with previous studies from our group showing that sialic acid type can modulate host-pathogen interactions and human-specific susceptibility to other infectious diseases such as typhoid fever and malignant malaria [21, 22], motivated us to investigate if the loss of Neu5Gc plays a role in human susceptibility to cholera. In this paper, we explore a variety of possible mechanisms and their potential link to the human-specific occurrence of cholera.

## Results

### Absence of Neu5Gc favors *V*. *cholerae* neuraminidase activity on complex gangliosides and enhancement of Ctx binding

**Fig 1:**
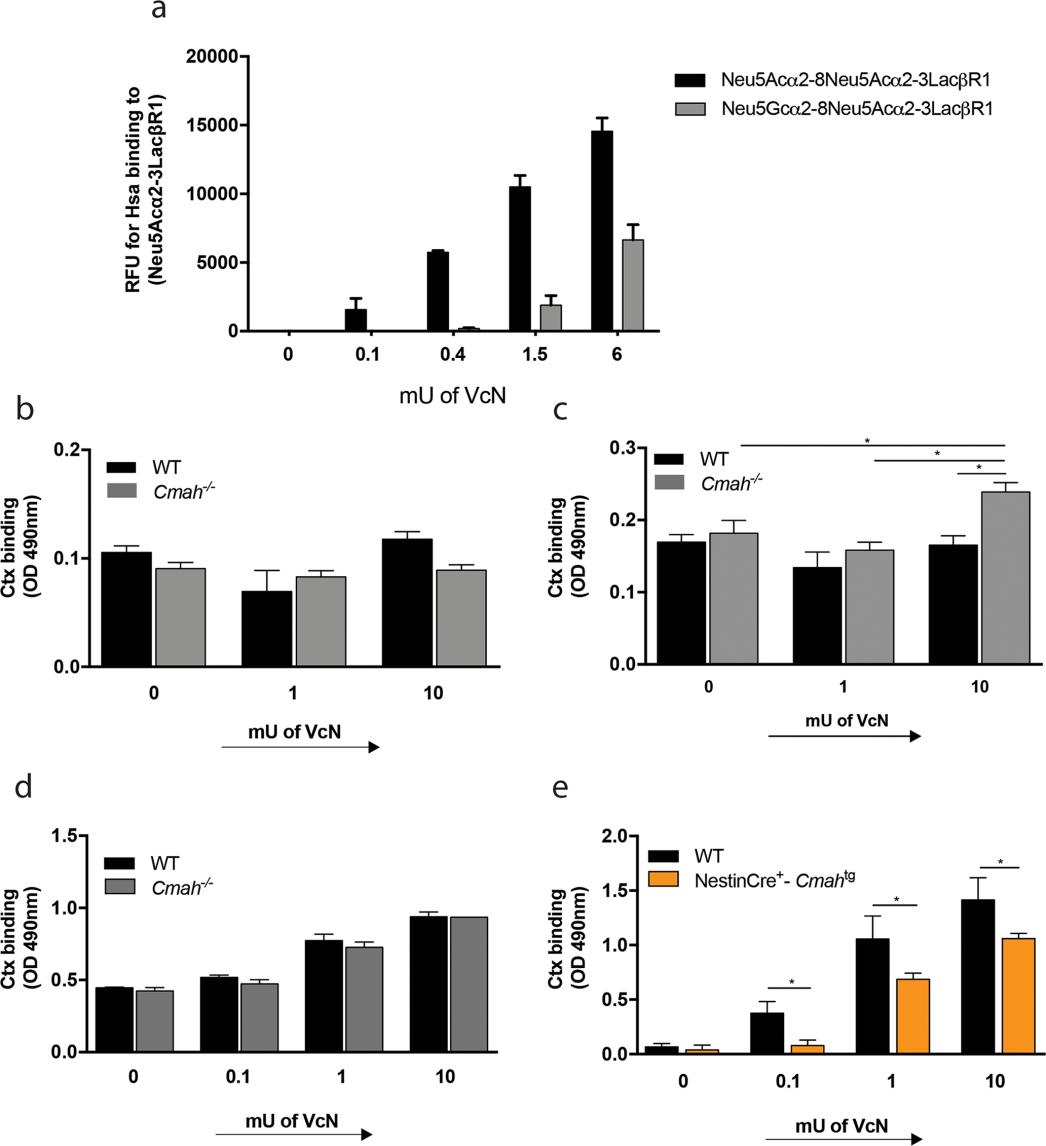
The presence of Neu5Gc compromises VcN activity and generation of epitopes for Ctx binding, compared to Neu5Ac. (a) Glass slides were printed with the indicated synthetic structures and incubated with increasing amounts of VcN. The resulting slides were probed with the bacterial Hsa-BR adhesin to detect exposed α2-3-linked mono sialic acid. Monosialogangliosides (b) and di-tritetrasialogangliosides (c) isolated from mice small intestine mucosa or from the brain (d and e) were treated with increasing amounts of VcN and incubated with 10ug/mL of biotin-conjugated B subunit of Ctx. Samples of gangliosides isolated from the brain of mice genetically engineered to express Neu5Gc in the brain [25] are referred as NestinCre^+^-*Cmah*^*tg*^ in figure (e). For all samples, Ctx binding was measured after incubation of the wells with HRP-Streptavidin and OPD substrate. The graphs are representative of three independent technical replicates using the batch of gangliosides isolated as described in the methods (*p<0.05).

*V*. *cholerae* neuraminidase (VcN) can catalyze the cleavage of α2-3- and α2-8-linked sialic acids from complex gangliosides with multiple sialic acids but it specifically does not cleave the single sialic acid α2-3-linked to an internal galactose (Gal) residue present in the GM1 ganglioside [4, 12]. VcN action on polysialogangliosides is thus critical to generate high concentrations of GM1 and favor the binding and internalization of Ctx by intestinal epithelial cells [4, 12]. Unrelated prior studies from our group have shown that glycans containing α2-8-linked Neu5Gc are relatively resistant to hydrolysis by other bacterial neuraminidases compared to glycans containing α2-8-linked Neu5Ac [20]. To study possible substrate preferences of VcN for α2-8-linked sialic acids, glycan structures containing terminal α2-8-linked Neu5Ac or Neu5Gc with underlying α2-3-linked Neu5Ac were exposed to increasing amounts of VcN and the resulting glycans were probed with Hsa, a bacterial adhesin that specifically recognizes any exposed α2-3-linked terminal mono-sialic acid [23]. As seen in Fig 1A, de-sialylation of Neu5Ac- or Neu5Gc-terminated sialoglycans with identical underlying glycan structures depended on VcN concentration. When the outermost α2-8-linked sialic acid moiety was removed by VcN, the underlying mono-sialic acid α2-3-linked to lactose became exposed and susceptible to Hsa binding. The Neu5Ac-terminated sialoglycan was more readily cleaved by VcN treatment than its Neu5Gc-terminated counterpart. Thus, VcN prefers Neu5Ac, and glycans containing α2-8-linked Neu5Gc are less susceptible to VcN-mediated hydrolysis (Fig 1A). Interestingly, when higher concentrations of VcN were used, it could further cleave the exposed terminal α2-3-linked Neu5Ac, resulting in a decrease of binding by Hsa, which specifically recognizes terminal α2-3-linked mono-sialic acids (S1 Fig).

Since Neu5Gc compromises VcN activity, we next tested if VcN treatment would influence the binding of Ctx to gangliosides isolated from WT and *Cmah*^*-/-*^ mice. ELISA assays revealed that treatment of small intestinal monosialogangliosides with increasing concentrations of VcN did not influence the binding of Ctx in samples from either mouse (Fig 1B). This was expected because VcN should not target monosialogangliosides to generate GM1 [24]. However, VcN treatment of di/tri/tetrasialo-gangliosides increased binding of Ctx to gangliosides from *Cmah*^*-/-*^ small intestine compared to gangliosides from WT mice (Fig 1C), suggesting that higher levels of GM1 were generated by VcN activity on complex gangliosides in the absence of Neu5Gc.

Even mammals that express *CMAH* in other tissues lack glycans containing Neu5Gc in their brain, a tissue enriched in gangliosides. Correspondingly, although VcN treatment of di/tri/tetrasialo-gangliosides isolated from the brain increased Ctx binding, the effect was comparable between WT and *Cmah*^*-/-*^ brain gangliosides (Fig 1D). Moreover, compared to WT controls, Ctx binding to brain gangliosides after VcN treatment was significantly lower in samples from NestinCre^+^-*Cmah*^tg^ mice that were genetically modified to overexpress Neu5Gc in the brain [25] (Fig 1E).

**Fig 2:**
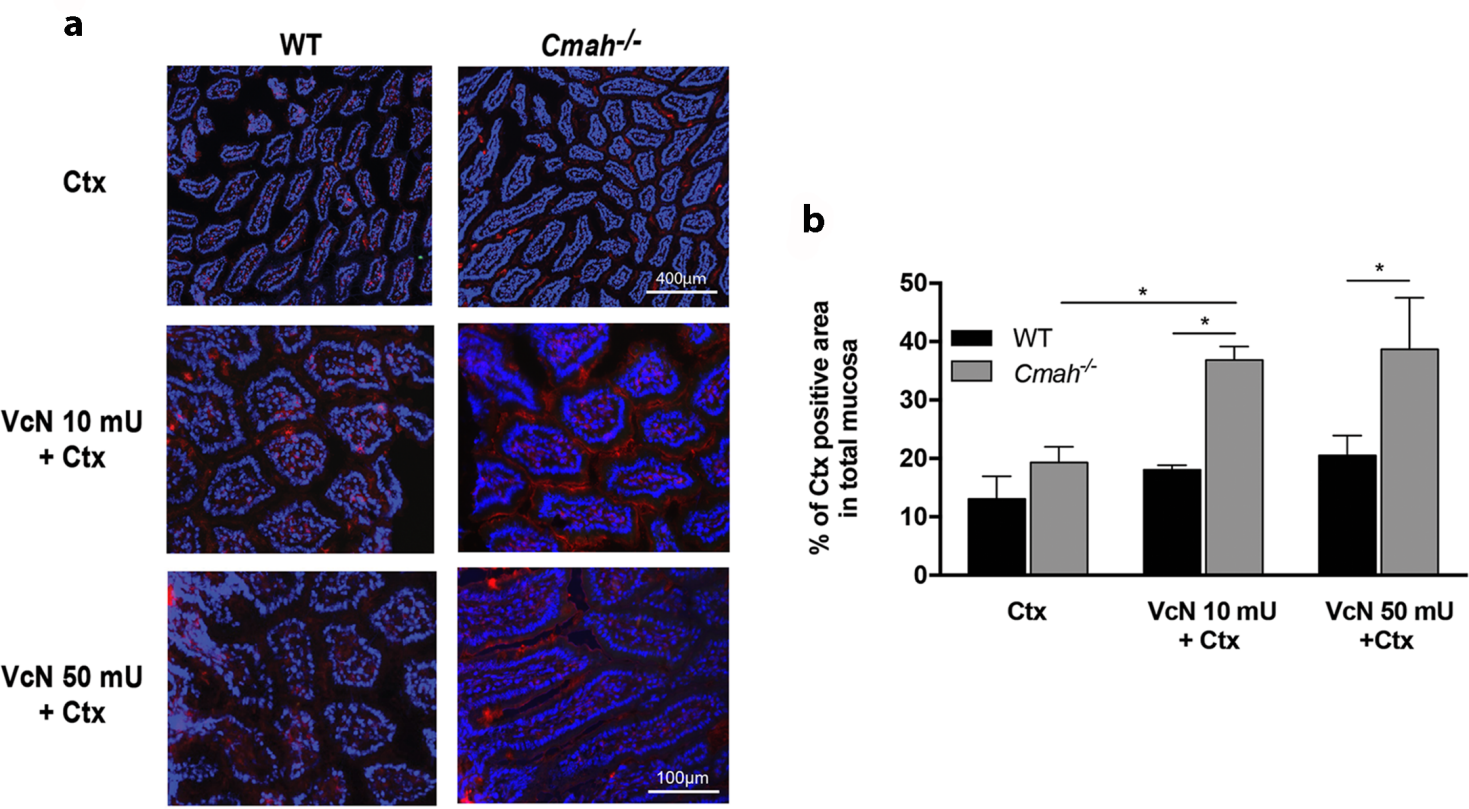
Ctx binding to small intestine frozen sections exposed to VcN is improved in the absence of Neu5Gc. (a) Fluorescence microscopy from frozen sections of small intestine from WT and *Cmah*^*-/-*^ mice incubated with biotin-conjugated B subunit of Ctx alone (top panels) or treated with 10 mU (middle panels) or 50 mU (bottom panels) of VcN. (b) Ctx binding was determined by incubation with Cy3-conjugated streptavidin and the Cy3 positive were quantified as Ctx binding area using imageJ. (*p<0.05).

To extend the findings from the ELISA assay, we compared the binding of Ctx to small intestinal tissue sections from WT and *Cmah*^*-/-*^ mice by microscopy. Although there was no significant difference in Ctx binding between WT and *Cmah*^*-/-*^ tissues at baseline (Fig 2A, top panels), treatment of frozen sections with VcN enhanced binding of Ctx to *Cmah*^*-/-*^ (Fig 2A, right panels) but not WT tissues (Fig 2A, left panels), as shown by the quantification of the Ctx-positive (red) area (Fig 2B). Together with our previous biochemical observation that absence of Neu5Gc facilitates VcN activity, these results suggest that higher levels of GM1 were generated in the tissue of *Cmah^-/-^* mice, thereby promoting Ctx binding to small intestinal gangliosides of *Cmah^-/-^* mice.

### VcN activity and Ctx-induced cAMP elevation are suppressed in human T84 cells expressing Neu5Gc

**Fig 3:**
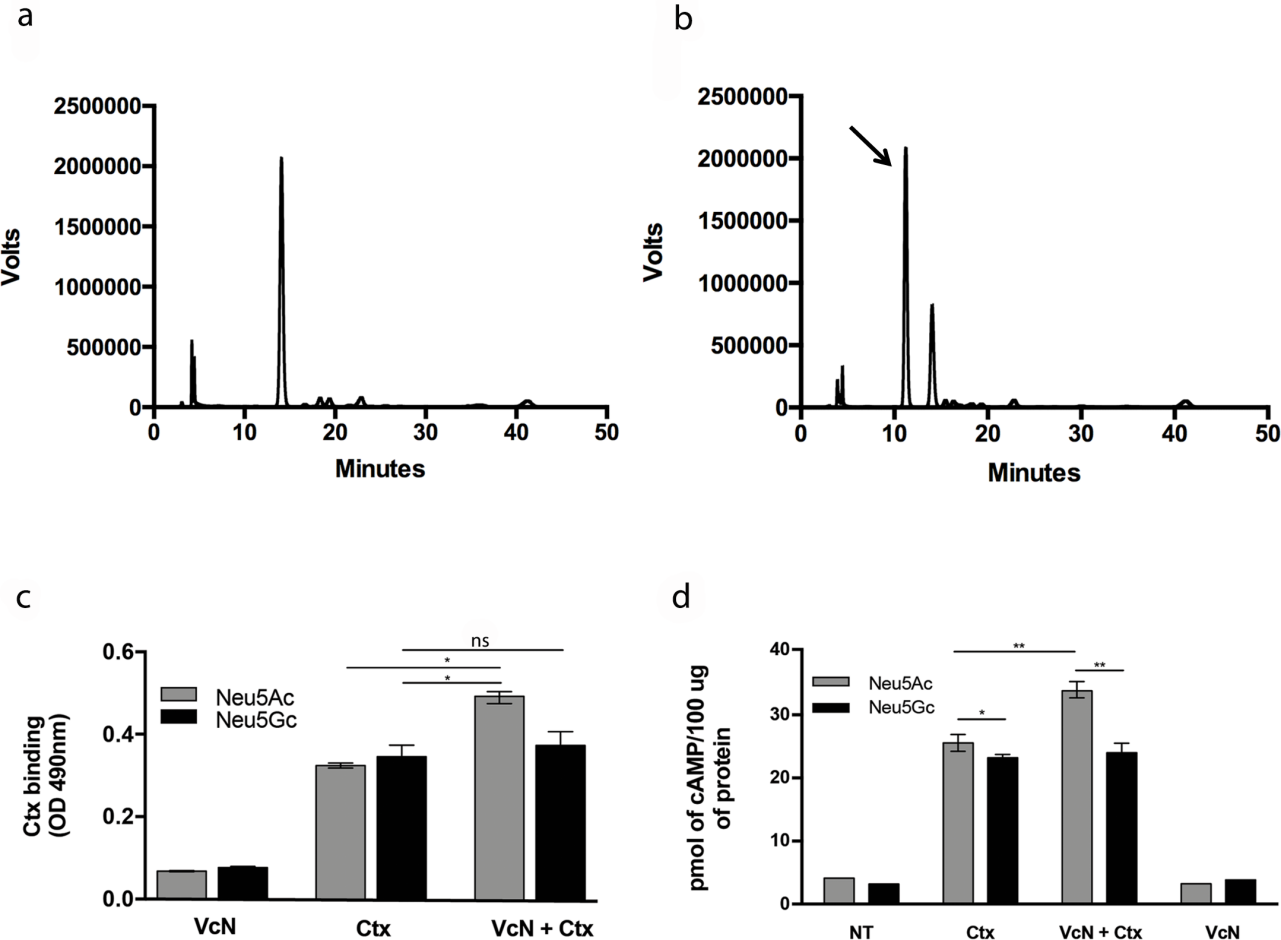
Human cells expressing Neu5Gc are resistant to VcN activity in generating Ctx binding and cell intoxication. (a-b) DMB-HPLC analysis for sialic acid incorporation in the cell surface glycans of T84 cells fed with 5mM of Neu5Gc (right histogram) or 5mM of Neu5Ac (left histogram) as a control. Arrow indicates the Neu5Gc-corresponding peak in Neu5Gc fed cells that is absent in Neu5Ac fed cells. NT = Not treated. (c) Whole cell ELISA (6 wells for each condition) for Ctx binding to T84 cells with or without VcN treatment. (d) Quantification of cAMP production induced by CtxAB5 with or without VcN treatment calculated for three different wells (biological triplicates) for each condition. Graphs represent one of three independent experiments. Black bars—Neu5Gc fed cells. Gray bars Neu5Ac expressing cells. (*p<0.05 and **p<0.005).

To confirm that absence of Neu5Gc plays a role in the pathophysiologic changes induced by VcN plus Ctx when *CMAH* is missing, we supplied exogenous Neu5Gc to human colonic cells in culture, which would be metabolically incorporated into cell surface glycoconjugates. We then analyzed VcN activity, Ctx binding and basal and stimulated cAMP levels in the cells that had metabolically incorporated Neu5Gc. As seen in Fig 3, T84 cells fed with free Neu5Gc (5 mM) successfully incorporate it into cell surface glycans (Fig 3B, black arrow). This result corroborates prior observations that human cells can incorporate Neu5Gc into nearly all major glycans, including gangliosides [17]. Importantly, cells fed with Neu5Ac, used as a control for sialic acid feeding, did not express detectable levels of Neu5Gc (Fig 3A). Using a whole cell ELISA assay [26], we observed that simply incorporating Neu5Gc in the cell surface did not by itself affect Ctx binding to T84 cells (Fig 3C). However, when cells fed with Neu5Ac were also exposed to VcN, binding of Ctx was significantly increased, whereas VcN had no effect on Ctx binding to cells fed with Neu5Gc (Fig 3C). Further, VcN significantly increased the ability of Ctx to elevate cAMP in cells fed with Neu5Ac- but not in Neu5Gc-fed cells (Fig 3D). Thus, lack of Neu5Gc makes glycans of human cells more susceptible to VcN activity in generating Ctx-binding and subsequent cell intoxication.

### Human-like *Cmah*^*-/-*^ mouse small intestine is more susceptible to Ctx-induced fluid secretion

**Fig 4:**
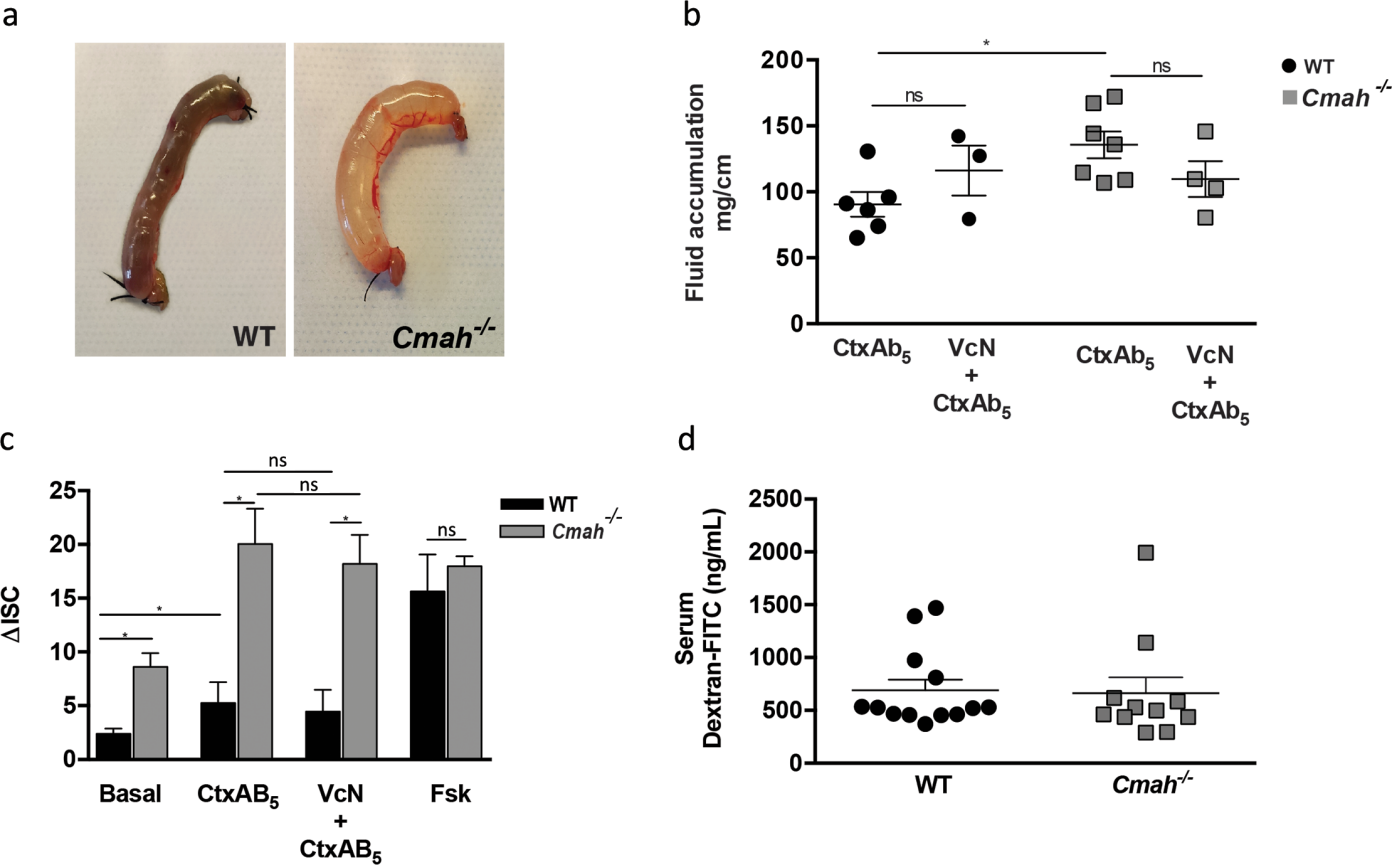
Increased susceptibility of *Cmah*^*-/-*^ small intestine to Ctx-induced fluid secretion. (a) Representative pictures of Intestinal loops of WT and *Cmah*^*-/-*^ treated with 100uL of PBS containing 10μg/mL of Ctx during 4 h. (b) Quantification of fluid accumulation in intestinal loops treated with 100uL of PBS containing 10μg/mL of Ctx plus and minus 10mU of VcN during 4 h, based on the loop weight/length ratio. (c) Ussing chamber measurement of ion secretion from small intestine fragments (black bars for WT and gray bars for *Cmah*^*-/-*^) exposed to 10ug/mL of active CtxAB5 for 4 h (n = 4) or Forskolin exposure (*p<0.05). (d) Measurement of FITC recovery in the serum of mice treated with FITC-dextran by oral gavage.

Since lack of Neu5Gc increased Ctx binding to the small intestinal gangliosides of *Cmah*^*-/-*^ mice, and exogenous incorporation of Neu5Gc blocked the ability of VcN to increase Ctx toxicity in human cells, we next studied pathogenic effects of both virulence factors in WT vs. *Cmah*^*-/-*^ mice. We hypothesized that WT mice that express Neu5Gc would be less susceptible to VcN-generation of GM1 from α2-8-sialylated gangliosides and thus develop fewer epitopes for Ctx binding. Conversely, *Cmah*^*-/-*^ mice should be more susceptible to Ctx-mediated fluid secretion because more GM1 would be readily generated from the α2-8-sialylated gangliosides due to the lack of Neu5Gc. Because adult mice have many compensatory mechanisms to prevent fluid loss, and therefore do not typically develop frank diarrhea following oral infection with *V*. *cholerae* (or many other non-invasive intestinal pathogens) [27–29], researchers have used ligated intestinal loops to study Ctx-induced pathogenesis [30, 31]. Using the same well-established model, we injected Ctx into intestinal loops created in WT and *Cmah*^*-/-*^ mice. After 4 h, Ctx induced fluid accumulation in both mice, but fluid levels were significantly higher in the intestinal loops of *Cmah*^*-/-*^ mice (Fig 4A and 4B). Corroborating this finding, incubation of jejunal segments with Ctx in Ussing chambers increased ion transport (most likely reflective of electrogenic chloride secretion) in *Cmah*^*-/-*^ specimens compared to that in WT tissue (Fig 4C). Basal ion transport was also upregulated in tissues from *Cmah*^*-/-*^ mice compared to WT, but there was no significant difference in ion secretion induced by forskolin between WT and *Cmah*^*-/-*^ tissues (Fig 4C). This latter finding implies that the enhanced ion transport response of *Cmah*^*-/-*^ tissues to Ctx is not simply due to differences in responsiveness of the ion transport machinery to intracellular cAMP. VcN addition to the intestinal loops or to the apical reservoir in the Ussing chamber did not affect Ctx-induced fluid (Fig 4B) or ion secretion across either WT or *Cmah*^*-/-*^ tissues (Fig 4C). However, unlike the effect of VcN treatment in the tissue sections, the apically-applied enzyme would face competitive inhibition by the heavy overlying layer of sialylated intestinal mucins and therefore could not reach the ganglioside structures. In natural human infection the bacterium may deploy other virulence mechanisms to reach the epithelial surface. Despite the lack of an efficient VcN-mediated desialylation process in this model, a significant increase in the fluid secretion from *Cmah*^*-/-*^ tissue nevertheless occurred, indicating that these mice are more susceptible to the action of Ctx itself, by an as yet unknown mechanism. In summary, our results show that the absence of glycans containing Neu5Gc makes the intestinal tissue of *Cmah*^*-/-*^ mice more susceptible to the pathogenic effects of Ctx.

It is well known that changes in the mucin expression profile in murine small intestine can alter tissue permeability [32, 33]. In addition, Ctx-mediated impairment of endosome recycling disrupts tight junction integrity and thus the intestinal barrier [10], which might enhance fluid loss. Because the mucins of WT and *Cmah*^*-/-*^ mice small intestine differ in their sialic acid composition, it was important to determine if the absence of Neu5Gc influenced intestinal permeability to account for increased Ctx-induced fluid secretion across *Cmah*^*-/-*^ small intestine. However, no differences in intestinal FITC-dextran permeability were seen between WT and *Cmah*^*-/-*^ mice (Fig 4D).

### Human-like absence of Neu5Gc in mice does not influence small intestinal colonization by *V*. *cholerae*

To deliver Ctx and induce diarrhea, *V*. *cholerae* must colonize the host small intestine in a process mediated by many different virulence factors [19, 34–37]. *V*. *cholerae* successfully colonizes the human small intestine but is unable to naturally colonize the intestines of other adult mammals studied to date [38]. In addition, Ctx-induced secretion of sialylated mucins plays an important role in *V*. *cholerae* colonization of the intestinal tract [39, 40]. Since mucins from *Cmah*^*-/-*^ mice mimic mucins of human tissues by completely lacking Neu5Gc, we analyzed whether the absence of Neu5Gc could influence colonization by *V*. *cholerae*. Although we found different levels of bacteria along the small intestine and colon of the infected mice, the bacterial CFU recovered from the small intestinal fractions were similar in both WT and *Cmah*^*-/-*^ mice (S2 Fig). These results suggest that the colonization of the small intestine of *Cmah*^*-/-*^ mice by *V*. *cholerae* is not significantly modulated by the absence of Neu5Gc. We next wanted to investigate *V*. *cholerae* growth in minimum media containing either Neu5Ac or Neu5Gc as sole carbon source and without competition from other bacteria. While *V*. *cholerae* growth was modestly sensitive to sialic acid differences at high concentrations (such as 3 mM), no differences in bacterial growth were observed at lower concentrations of the two monosaccharides (S3 Fig).

Although similar levels of *V*. *cholera* were recovered from the gut tissues of both WT and *Cmah*^*-/-*^ mice, we asked if increased susceptibility of *Cmah*^*-/-*^ small intestine to Ctx-induced fluid secretion (Fig 4A, 4B and 4C) would make those animals develop diarrhea after ingesting live *V*. *cholerae*. Neither WT nor *Cmah*^*-/-*^ *mice* gavaged with 2 × 10^8^ CFU of *V*. *cholerae* showed any signs of diarrhea. Thus, although the lack of Neu5Gc increases susceptibility to Ctx toxicity, the *Cmah*^*-/-*^ background is not sufficient in itself to make mice susceptible to frank experimental diarrhea induced by oral infection with *V*. *cholera*. These results corroborate previous studies that have shown that adult mice do not develop diarrhea following oral infection with *V*. *cholerae*, although the bacteria are able to replicate in the host gut (S2 Fig) [41].

## Discussion

There are multiple potential mechanisms by which the human evolutionary loss of epithelial Neu5Gc could contribute to the human-specific susceptibility to cholera. These include improved survival in gastric fluid (not studied here); colonization or preferential growth with Neu5Ac or Neu5Gc as carbon source (no effect seen here); decreased VcN degradation of inhibitory mucin sialic acids (not studied here); VcN remodeling of higher gangliosides into GM1 (a major impact seen here); improved delivery of the CtxA subunit into the cytosol (not studied here); and cAMP production and chloride channel activation (both shown here to be markedly enhanced). While some hypotheses were nullified, we discovered more than one likely contributory mechanism that could underlie the human-specific susceptibility to cholera.

We demonstrated that VcN shared its substrate preference with other previously studied mammalian and bacterial sialidases [20], in being much less able to cleave α2-8-linked Neu5Gc than α2-8-linked Neu5Ac, which also corroborates another recent report [42]. We additionally showed that higher amounts of VcN are required to cleave an inner α2-3-linked sialic acid after removal of the outer α2-8-linked sialic acid, indicating that VcN has a preference for α2-8- over α2-3-linked sialic acids. Previous reports showed that VcN hydrolyses α2-3-sialyl galactoside more efficiently than α2-6-sialyl galactoside [43, 44]. Taken together, we theorize that *V*. *cholerae* neuraminidase evolved to target human gangliosides preferentially, which are terminated only by Neu5Acα2-8-Neu5Acα2-3Gal1- and/or Neu5Acα2-3Galβ1-linked sialic acids. Nonetheless, the presence of α2-8-linked Neu5Gc on gangliosides compromised VcN hydrolysis, which fits our findings concerning the increased generation of GM1 binding sites for Ctx in the small intestine of *Cmah*^*-/-*^ mice. Our results also show that VcN treatment affects the binding of Ctx to gangliosides isolated from the small intestine and brain of *Cmah*^*-/-*^ and NestinCre^+^-*Cmah*^*tg*^ mice, demonstrating that gangliosides lacking Neu5Gc are better targets for VcN to generate GM1 from a mixture of complex gangliosides. It is worth mentioning that the differences in Ctx binding observed in our experiments should solely correlate with the amount of GM1 generated after VcN action, since Ctx itself binds equally to either Neu5Gc-GM1 and Neu5Ac-GM1 [16].

Human cells induced to express glycans containing Neu5Gc are less susceptible to VcN activity, and therefore permit less generation of the optimal GM1 target for Ctx. The loss of Neu5Gc in humans confers increased susceptibility to VcN, which in turn favors Ctx-induced toxicity in intestinal cells. However, an additional mechanism evidently makes the small intestine of human-like *Cmah*^*-/-*^ mice more susceptible to Ctx-induced fluid secretion.

Early studies using germ-free mice demonstrated that the mouse gut microbiota is a natural impediment to *V*. *cholerae* colonization [45]. Because of this, many studies have used oral treatment with streptomycin as a colon dwelling model to study intestinal infection by this bacterium [46, 47]. In fact, providing streptomycin in the drinking water did allow the colonization of the gastrointestinal tract of both WT and *Cmah*^*-/-*^ mice by *V*. *cholerae*. Additional studies have shown that the catabolism of sialic acid initiated by VcN is an important step for intestinal colonization and the pathogenesis of cholera [48] and that *V*. *cholerae* can grow *in vitro* when sialic acid is used as the sole carbon source [49]. Here, we show that *V*. *cholerae* colonizes the small intestine of WT and *Cmah*^*-/-*^ mice similarly, corroborating previous data that the bacteria can use both Neu5Ac and Neu5Gc as nutrient sources [49].

During natural infection, live bacteria depend critically on their toxin-coregulated pilus (TCP) structure to colonize the human small intestine [34–36]. The action of TCP helps the bacteria to get through the mucin barrier [50] and facilitates their direct contact with the epithelial surface where VcN and Ctx act locally to induce diarrhea. We suspect that when purified VcN is injected into the lumen, it heavily engages the highly sialylated mucin layer overlying the epithelial surface and therefore does not reach the relevant ganglioside structures to generate GM1. This could explain why treatment with VcN did not potentiate the effect of Ctx in tissue segments mounted in Ussing chambers. In keeping with this rationale, VcN treatment did increase Ctx binding to frozen sections of the small intestine, wherein the mucus barrier is bypassed by tissue sectioning.

**Fig 5:**
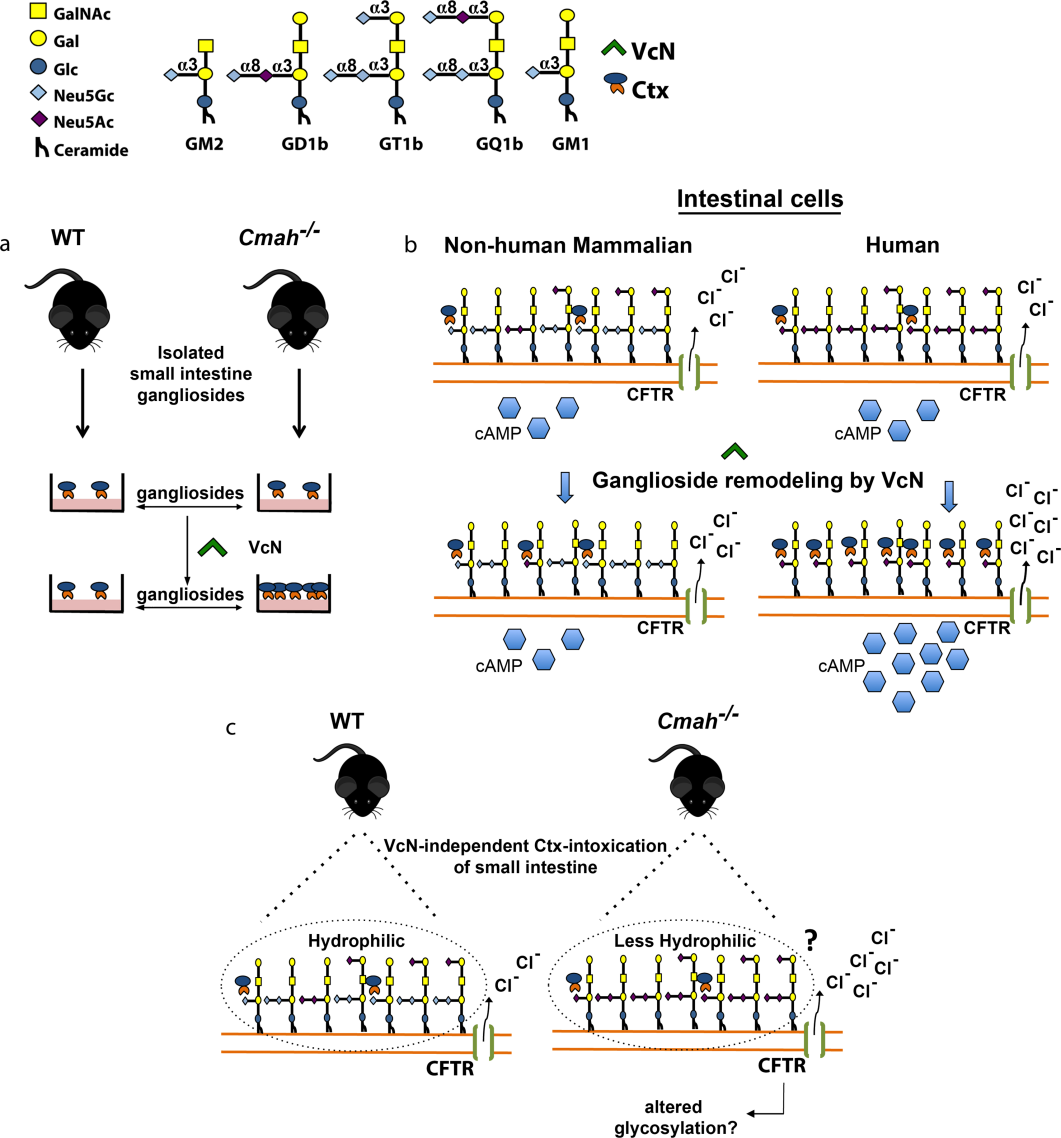
Schematic representation of VcN-mediated remodeling of mucosal complex gangliosides to GM1 and possible VcN-independent mechanisms that lead to increased susceptibility to Ctx intoxication in the absence of Neu5Gc. VcN cleaves α2–8 and α2-3-linked sialic acids from di-, tri-, and tetra-sialylated gangliosides to generate the monosialylated ganglioside, GM1. Importantly, VcN is not able to cleave the α2-3-linked sialic acid in the GM1 and thus it the optimal target for Ctx is retained. Di-, tri-, tetra-sialylated gangliosides from WT mice (containing α2-8-linked Neu5Gc) are resistant to VcN cleavage and thus much less GM1 is generated (a—left panels). In contrast, VcN more efficiently cleaves di-, tri-, and tetra-sialylated gangliosides from *Cmah*^*-/-*^ mice, resulting in increased binding of Ctx (a—right panels). In keeping with this rationale, the remodeling of human gangliosides to GM1 that is catalyzed by VcN (b—right panels) occurs at much higher levels than in non-human mammalian cells (b—left panels). We show here that there is also an unknown VcN-independent mechanism by which the small intestine of *Cmah*^*-/-*^ mice is more susceptible to Ctx intoxication. We speculate that the increased levels of ion secretion across the small intestine of *Cmah*^*-/-*^ mice produced by Ctx also rely on changes in the hydrophobic/hydrophilic properties of the cell surface glycocalyx (c). Epithelial cells surfaces may contain up to 10^8^ residues of sialic acids. Thus, the abundance of hydroxyl groups in Neu5Gc-positive cells increases the hydrophilicity of their glycocalyx. On the other hand, the glycocalyx of human cells should be less hydrophilic since it only contains glycans with Neu5Ac. Although not studied here, it is also possible that CFTR glycosylation differs in the absence of Neu5Gc, perhaps modulating its trafficking to the cell suface in response to Ctx (c).

A recent study elegantly showed that human T84 cells have little or no GM1 and that fucosylated and sialylated glycoproteins are instead the main target for Ctx binding to those cells [26]. Follow up studies further demonstrated that Ctx binds to fucosylated Le^x^-carrying glycoproteins but not to Le^x^-carrying glycolipids and that human and mouse intestinal cells can be intoxicated by Ctx, even when glycosphingolipid synthesis in inhibited [51]. Although these very interesting findings shed new light in the understanding of additional binding sites for Ctx, which are likely to also be relevant in disease pathogenesis, the authors did not study the impact of VcN in the Ctx binding to glycoproteins. We hypothesize that VcN would hydrolyze α2-3- and α2-6-linked sialic acid from glycoproteins to prevent Ctx binding to such molecules. VcN would also degrade di-tri sialylated gangliosides to generate GM1 and Ctx binding (See Fig 5). Together with the above arguments, our data suggest that intestinal cells with Neu5Gc-containing gangliosides are less susceptible to the toxicity of Ctx, and that this occurs at least in part due to the differential action of VcN on Neu5Gc compared to Neu5Ac-containing ganglioside targets.

Although the data generated using the mouse model neither supported nor ruled out the hypothesis that VcN action enhanced the generation of GM1 target in isolated gangliosides, we found the small intestine of *Cmah*^*-/-*^ mice secreted much higher levels of fluid in response to Ctx_,_ suggesting additional mechanisms by which loss of Neu5Gc increases host susceptibility to Ctx. Fluid secretion results from both intense Cl^-^ secretion through CFTR reflected as changes in short circuit current [52] as well as inhibition of NaCl absorption, which cannot be assessed in Ussing chambers. Glycosylation of CFTR is crucial for its function, affecting its cell surface expression, conformation stability and lysosomal degradation [53]. CFTR in *Cmah*^*-/-*^ mice will only contain glycans with Neu5Ac, unlike CFTR glycans in WT mice (and adult mammals other than humans) that would express both Neu5Gc and Neu5Ac. We speculate that the differences in CFTR glycosylation could influence the increased electrogenic ion transport across *Cmah*^*-/-*^ small intestine as observed in Ussing chambers, and also the intense fluid secretion in the *Cmah*^*-/-*^ intestinal loops.

Enterotoxigenic E. coli (ETEC) produce a heat labile enterotoxin (LT), which is ca. 80% identical to Ctx, and acts by a similar mechanism also binding to GM1 [54]. Yet ETEC infects both humans and animals and does not produce a neuraminidase. With ETEC, the difference between strains that infect animals and strains that infect humans is may rely on both enterotoxin and intestinal colonization factors such as CFAI or K88 [54] that confer species specificity in binding of the whole bacterium. At first glance the situation with ETEC would seem to argue against our conclusions that the VcN specificity and differences in intestinal sialic acids accounts for *V*. *cholerae* being a human-specific pathogen. However, there is a major difference between the pathogens that needs to be considered. Whereas ETEC LT can only bind to the very small amount of GM1 naturally present in the gut mucosal epithelia, VcN provides extensive trimming of the complex gangliosides, maximizing the production of a much larger amount of GM1 target that, as a striking coincidence, is completely resistant to further VcN cleavage (See Fig 5). Indeed, this difference can help explain the much greater severity of the diarrhea in terms of volume loss in cholera than in ETEC infections. We do not rule out the possibility that the human specificity of cholera may also include other components such as species-specific intestinal colonization factors.

Taken together, our results show that the evolutionary loss of epithelial Neu5Gc is a potential mechanism that contributes, at least in part, to the human-specific susceptibility to cholera, and can explain what appears to be one of the rare instances in which Robert Koch was unable to fulfill his famous postulates [18] for a human infectious disease: isolate the organism from the natural host, grow it in culture and introduce it back into an animal model host to reproduce the disease.

## Materials and methods

### Reagents

T84 cells (American Type Culture Collection—CCL-248), a line derived from a lung metastasis of a human colorectal carcinoma, were used as previously described [55]. The active (AB_5_) form of Ctx, 4 kDa labeled FITC-dextran and 1,2-diamino-4,5-methylenedioxybenzene (DMB) were obtained from Sigma-Aldrich (St. Louis, MO, USA). *V*. *cholerae* neuraminidase was purchased from Roche (Clovis, CA, USA). The cAMP ELISA kit was from R&D Systems (Minneapolis, MN, USA). *V*. *cholerae* strain N16961 was acquired from ATCC (Manassas, VA, USA).

### Determination of *V*. *cholerae* neuraminidase substrate preference by a pair of Neu5Ac- vs. Neu5Gc-terminated glycans

The pairs of glycans [56] were printed on glass slides. Resulting slides were blocked with ethanolamine, washed, dried, and then fitted into a hybridization cassette [21, 23]. The slides were then blocked with ovalbumin (1% w/v) in PBS (pH 7.4) for 1 h at RT, with gentle shaking. Subsequently, various amounts of VcN were applied to the glass slides. After incubating for 2 h, the slides were extensively washed to remove the neuraminidase. A GST-tagged bacterial adhesin probe, Hsa, which specifically recognizes mono sialic acid α2-3-linked to underlying glycan structures [23] was applied and incubated for 2 h at RT with gentle shaking, followed by anti-GST antibodies (1:2000 dilution in DPBS) and Alexa Fluor 555-conjugated anti-rabbit IgG (1:10.000 in DPBS) incubation. Resulting slides were washed and dried. Finally, the slides were scanned with a Genepix 4000B scanner (Molecular Devices Corp., Union City, CA). Data were analyzed with the Genepix Pro 7.0 analysis software.

### Mice

*Cmah*^*−/−*^ mice, generated as previously described [57], were bred onto a congenic C57BL/6 background and maintained in the University of California, San Diego vivarium according to Institutional Animal Care and Use Committee (IACUC) guidelines. *Cmah*^*−/−*^ and WT mice were raised in the same vivarium room, in the same cage rack and fed with the same chow and water source to avoid variations in the gut microbiome. Both male and female mice from 8–14 weeks of age were used for all experiments.

### Ethics statement

All studies were approved by the UC San Diego Institutional Animal Care and Use Committee (IACUC) under the protocol number S01227. All studies complied with federal regulations regarding the care and use of laboratory animals: Public law 99–158, the Health Research Extension Act, and Public Law 99–108, the Animal Welfare Act which is regulated by USDA, APHIS, CFR, Title 9. Parts 1, 2, and 3.

### Ganglioside purification

Mice were euthanized in the humane CO_2_ chamber according to our animal protocol approval and the small intestine of WT and *Cmah*^*-/-*^ mice and brain tissues from WT, *Cmah*^*-/-*^ and NestinCre^+^-*Cmah*^tg^ [25] were harvested. Brains were bisected in the sagittal plane and the small intestine mucosa was exposed using a blunted pair of scissors. The mucosa was then harvested using a cell scraper. Approximately 4 g of small intestinal mucosa and 0.5 g of brain tissue were homogenized in 4 volumes of ice-cold water using a polytron homogenizer and a total lipid extraction from both tissues was performed as previously described [58]. Briefly, gangliosides from both tissue preparations were purified from neutral glycolipids by running the total lipid phase through a DEAE-Sephadex (Bio-rad) column as previously described [59]. Next, the fractions containing monosialogangliosides were eluted from the column using 0.01 M ammonium acetate and the di/tri/tetrasialo-gangliosides were eluted with 0.5M ammonium acetate. Gangliosides in each fraction were quantified using DMB derivatization and HPLC analysis as previously described [17] and the concentration of sialic acid was used to normalize for the ganglioside concentration.

### ELISA for Ctx binding to purified gangliosides

96 well plates (Cat: 9018 –Corning, USA) were coated with 100 μL of 0.2 nM of gangliosides purified from mouse brain and small intestine and left to dry overnight at RT. On the next day, the wells were washed 3 times with PBS and incubated with increasing amounts of VcN diluted in PBS for 45 min. The wells were washed 3 times with PBS and blocked with PBS containing 5% bovine serum albumin (BSA) for 2 h at RT. The blocking solution was aspirated from the wells and 100 μL of 40 μg/mL biotin-conjugated Ctx B subunit were added for 45 min. The wells were washed 3 times with PBS and 100μL of 1:5000 dilution HRP-streptavidin was added for 45 min. The wells were washed 3 times and 140 μL of *O*-phenylenediamine dihydrochloride (OPD) solution was added for approximately 10 min until the reaction was stopped with 40 μL of 4M H_2_SO_4_ solution and read at 490 nm.

### Fluorescence microscopy for Ctx binding to the small intestine

Samples of jejunum from freshly harvested WT or *Cmah*^*-/-*^ mouse small intestine, were flash frozen in optimal cutting temperature (OCT) and dry ice/isopentane slurry. Frozen sections were prepared at 5 μm and briefly air-dried, followed by blocking of endogenous biotin and then overlaid with 10 μg/mL of biotin-conjugated B subunit of Ctx either alone, or together with either 10 or 50 mU/mL of VcN, both diluted in PBS containing Ca^2+^ and Mg^2+^, for 45 min at RT. Following washes in PBS, the slides were incubated for 45 min with a 1:500 dilution of Cy3-conjugated streptavidin and then the nuclei were counter stained with Hoecsht. Digital images were captured using a Keyence BZ9000 using 10x and 40x magnification. Frozen sections from the small intestines of 4 WT and 4 *Cmah*^*-/-*^ mice were used for the fluorescence overlay assay using the following number of sections for each condition: Ctx (WT n = 5; *Cmah*^*-/-*^ n = 4); VcN 10 mU + Ctx (WT and *Cmah*^*-/-*^ n = 5); VcN 50 mU + Ctx (WT and *Cmah*^*-/-*^ n = 4). The percentage area per picture showing binding with Ctx was calculated using the formula (% of total fluorescent area—% Hoechst fluorescent area = % of Ctx positive area) using ImageJ 1.50i software. The observer who captured the pictures was blinded for the experimental groups.

### Ligated intestinal loop

To study the effect of VcN and Ctx on fluid secretion, mouse small intestinal loops were studied as previously described [10, 30, 31]. Briefly, WT and *Cmah*^*-/-*^ mice were anesthetized with a single intraperitoneal injection of 100 μL of a ketamine (70 mg/kg) + xylazine (10 mg/kg) solution in PBS. A midline laparotomy was performed and one ligated intestinal loop (≈ 3 cm) was formed in the jejunum of each animal using 5–0 USP 12 mm sutures. 100 μL of PBS containing 10 μg/mL of the active form of Ctx (AB_5_ Ctx) plus or minus 10mU of VcN were injected into the loop. The ligated loops were returned to the abdominal cavity, and the mice were kept anesthetized for 4 h with the body temperature kept at 37°C using heating pads. The animals were then euthanized in a CO_2_ chamber and the intestinal loops were excised and weighed to determine fluid accumulation, which was expressed as mg per cm of loop length.

### Ussing chamber analysis

An electrophysiological analysis of electrogenic ion transport across mouse intestinal segments was performed using an Ussing chamber system (Physiologic Instruments Inc., San Diego, CA, USA) as previously described [60]. Segments of jejunum from WT or *Cmah*^*-/-*^ mice were mounted in Ussing chambers (window area: 0.1 cm^2^) and both apical and basolateral hemichambers were filled with Ringer’s bicarbonate buffer containing (in mM): 140 NaCl, 25 NaHCO_3_, 2.4 KH_2_PO_4_, 0.8 K_2_HPO_4_, 1.2 CaCl_2_, 0.8 MgCl_2_ (pH 7.4). 10 mM mannitol or 10 mM glucose were added to the apical and basolateral hemichamber buffers, respectively. The solution was continuously bubbled with 5% CO_2_/95% O_2_ and maintained at 37°C. Tissues were voltage-clamped to zero potential difference by the application of short-circuit current (Isc), and a baseline was established as previously described [60]. Ctx (10 μg/mL) and/or VcN (10 mU/mL) were then added to the apical hemichambers and changes in Isc (ΔIsc), reflective of electrogenic chloride secretion, were measured at 4 h after treatment. Tissues from both mouse strains were also treated with forskolin (20 μM) and the ΔIsc was calculated at the time of peak response.

### Measurement of intestinal permeability *in vivo*

To compare intestinal permeability in WT and *Cmah*^*-/-*^ mice, both strains were orally gavaged with 44 mg of 4 kDa FITC-dextran per 100 g of body weight. The flux of FITC-dextran from the intestine to the bloodstream was determined 4 h after gavage by spectrophotofluorometry with 485 nm excitation and 528 nm emission wavelengths as previously described [61, 62].

### HPLC analysis for Neu5Gc incorporation into cell surface glycans of T84 cells

T84 cells were maintained in 1:1 DMEM-F12 medium supplemented with 5% newborn calf serum and 1% penicillin/streptomycin solution at 37°C and 5% CO_2_. To study the ability of these cells to incorporate Neu5Gc, 5 mM free Neu5Gc (INALCO) was added to the medium for three days using the same culture conditions. The incorporation of sialic acid into cell surface glycans was confirmed by DMB-HPLC analysis as previously described [17]. Briefly, the cells were detached with PBS containing 0.5 mM EDTA and submitted to hypotonic lysis in 0.1X diluted in water with three cycles of freezing (-70°C) and thawing (37°C). The resulting lysate was centrifuged at 25,000 RPM for 15 min and the pellets containing the cell membrane fractions were subjected to acid hydrolysis with 0.1 M H_2_SO_4_ at 80°C for 1 h to release sialic acid bound to cell membranes. After hydrolysis, the samples were centrifuged at 25,000 RPM for 15 min and the supernatant was filtered through an Amicon 10K column and derivatized in DMB reagent for 150 min at 50°C. The incorporation of Neu5Gc was analyzed by HPLC as previously described [17].

### In cell ELISA for Ctx binding to T84 cells

To investigate if Neu5Gc-containing glycans would influence Ctx binding, 25,000 T84 cells per well were seeded in 96-well (Cat: 353072 –Falcon, USA) plates and exposed to 5 mM free Neu5Ac or Neu5Gc for three days as described above. On the third day, the binding of Ctx to T84 cells was performed as previously described [26] with some modifications. Cells were washed 2 times in RT PBS and further incubated with 10mU of VcN in PBS pH 6 for 30 min at RT. The wells in the same plate that were not treated with VcN were kept in PBS at pH 6 in parallel. After VcN treatment, the wells were washed twice in cold PBS and incubated with 5 μg/mL of biotinylated Ctx B-subunit (CTB) in PBS (containing 1 mM CaCl_2_, 1 mM MgCl_2_, 0.2% (w/v) BSA, and 5 mM glucose) for 20 min on ice. Unbound biotin-CTB was removed by washing 3 times in cold PBS and the cells were fixed with 4% paraformaldehyde for 10 min on ice and 20 min at RT. After 3 washes with PBS, cells were blocked with 1% BSA/PBS for 45 min and incubated with HRP-streptavidin (Roche) at 1:10.000 dilution in PBS. HRP activity was measured by adding *ortho*-phenylenediamine (OPD) substrate and reading at 490 nm in a microplate reader (Molecular Devices). All values were corrected by light absorbance at 650 nm and normalized to total cell protein content (bicinchoninic acid assay, Pierce BCA protein assay kit, Pierce).

### cAMP measurement

To study whether Neu5Gc influences the activity of VcN and Ctx on intracellular levels of cAMP, 1 × 10^5^ T84 cells per well were seeded in 12-well plates (Cat: 353043—Corning Inc, USA) and treated for 3 days with 5 mM free Neu5Gc as described previously [63]. Cells fed with 5mM Neu5Ac were used as controls. After 3 days, fresh medium with no sialic acid was added. Cells were then treated with Ctx (10 μg/mL) in the presence or absence of VcN (10 mU/mL) for 6 h at 37°C and 5% CO_2._ The cells were washed twice with ice-cold PBS and the lysate was used to measure cAMP according to the manufacturer’s instructions. In parallel, the percentage of cell surface Neu5Gc vs. Neu5Ac was measured by DMB-HPLC on the third day after feeding as described elsewhere [64].

### Oral infection with *V*. *cholerae* El Tor N16961

For intestinal tissue colonization, *V*. *cholerae* El Tor serovar O:1 strain N16961 was grown overnight at 37°C from a -80°C frozen glycerol stock in Luria-Bertani broth (LB) medium. The next day, the overnight culture was centrifuged at 4000 rpm for 5 min and the pellet resuspended in 300 mM sodium bicarbonate (NaHCO_3_) buffered solution, pH 9.0. As per the streptomycin-treated model of cholera infection [47], mice were treated with 5 mg/mL of streptomycin (Sigma) in their drinking water one day before infection. The concentration of streptomycin was then lowered to 0.25 mg/ml for the duration of the experiment. Each animal received 1×10^8^ CFU in 100 μL of the above NaHCO_3_ buffer via oral gavage. At each time point, animals were humanely euthanized in CO_2_. The small intestine and colon were excised from the mice and the small intestine was divided into duodenum, jejunum, and ileum. Intestinal sections were opened lengthwise and stool was gently removed by washing in sterile PBS. Intestinal tissue was then cut into small segments and homogenized. Cholera CFU were enumerated by serial dilution and plating on Thiosulfate-citrate-bile salts-sucrose agar (TCBS) media.

### *In vitro* growth curve of *V*. *cholerae* in M9 minimal media containing different sialic acids

For *in vitro* growth curves, *V*. *cholerae* El Tor serovar O:1 strain N16961 was grown overnight as per oral infection. The overnight bacteria culture was diluted the next day at 1:20 into fresh LB medium and grown to early log phase at 37°C, corresponding to an optical density at 600 nm (OD_600 nm_) of 0.4. One mL of bacterial suspension was then spun down at 13,200 rpm for 5 min, washed and resuspended in M9 minimal media comprised of Na_2_HPO_4_ (6 g/L), KH_2_PO_4_ (3 g/L), NaCl (0.5 g/L), NH_4_Cl (1 g/L), MgSO_4_ (2 mM), and CaCl_2_ (0.1 mM). Bacteria (10 μL) were then added to 1 mL of M9 minimal media containing 0.2% (w/v) glucose, as well as M9 minimal media with 0.2% glucose plus Neu5Ac or Neu5Gc (3 mM, 1.5 mM, 0.75 mM, or 0.375 mM). Each inoculated media (200 μL) was added, in quadruplicate, to a Honeycomb plate and covered with a lid. Bacteria were then grown at 37°C for 24 h on a Bioscreen C (Bioscreen Inc). Growth was monitored by OD_600 nm_ readings at 30 minute intervals, and the growth curve was analyzed using GraphPad Prism version 7.

### Statistical analysis

Statistical analyses for two-group comparisons were performed using an unpaired Student’s t test. Analyses for multiple group comparisons were done using one-way ANOVA and Bonferroni post-test. Both analyses were performed using GraphPad Prism version 7. The data are represented as means ± standard deviation (SD) and P values of <0.05 were considered to represent statistically significant differences.

## Supporting information

S1 Fig Hydrolysis of α2-3-linked sialic by high amounts of VcN. Glass slides printed with the indicated synthetic structures were incubated with high amounts of VcN. The resulting structures were probed with bacterial Has adhesin to evaluate the cleavage of **α**2-3-linked mono sialic acid.
(TIF)

S2 Fig *V. cholerae* colonization of mice small intestine and colon measured by bacterial recovery. WT and *Cmah*^*-/-*^ mice were treated with 5mg/mL of streptomycin for 24 h prior to oral infection and with 0.25mg/mL of streptomycin during the remaining experiment time, both in the drinking water. The animals were infected with 2x10^8^ CFU’s of *V*. *cholerae* and small intestine fractions divided into jejunum (a), ileum (b) duodenum (c), and the colon (d) were homogenized for Cholera CFU counts after serial dilutions were plated on (TCBS) agar media. CFU recovery was determined in tissue fragments harvested from days 1, 2 and 3 after infection.
(TIF)

S3 Fig *V*. *cholerae* growth is modestly affected by sialic acid differences. Bacterial growth in minimal M9 medium supplemented with 0.2% of glucose and concentrations of 3mM (a) 1.5 mM (b) 0.75mM (c) and 0.0375mM (d) of Neu5Ac or Neu5Gc was measured at each 30 min interval during 24h using Bioscreen C system. Data is representative of three independent experiments, with each condition done in quadruplicate.
(TIF)
