# Supplementary figures and images for: Human evolutionary loss of epithelial Neu5Gc expression and species-specific susceptibility to cholera
Source: PLoS Pathog. 2018 Jun 18;14(6):e1007133. doi: 10.1371/journal.ppat.1007133 (PMC6023241; doi:10.1371/journal.ppat.1007133)

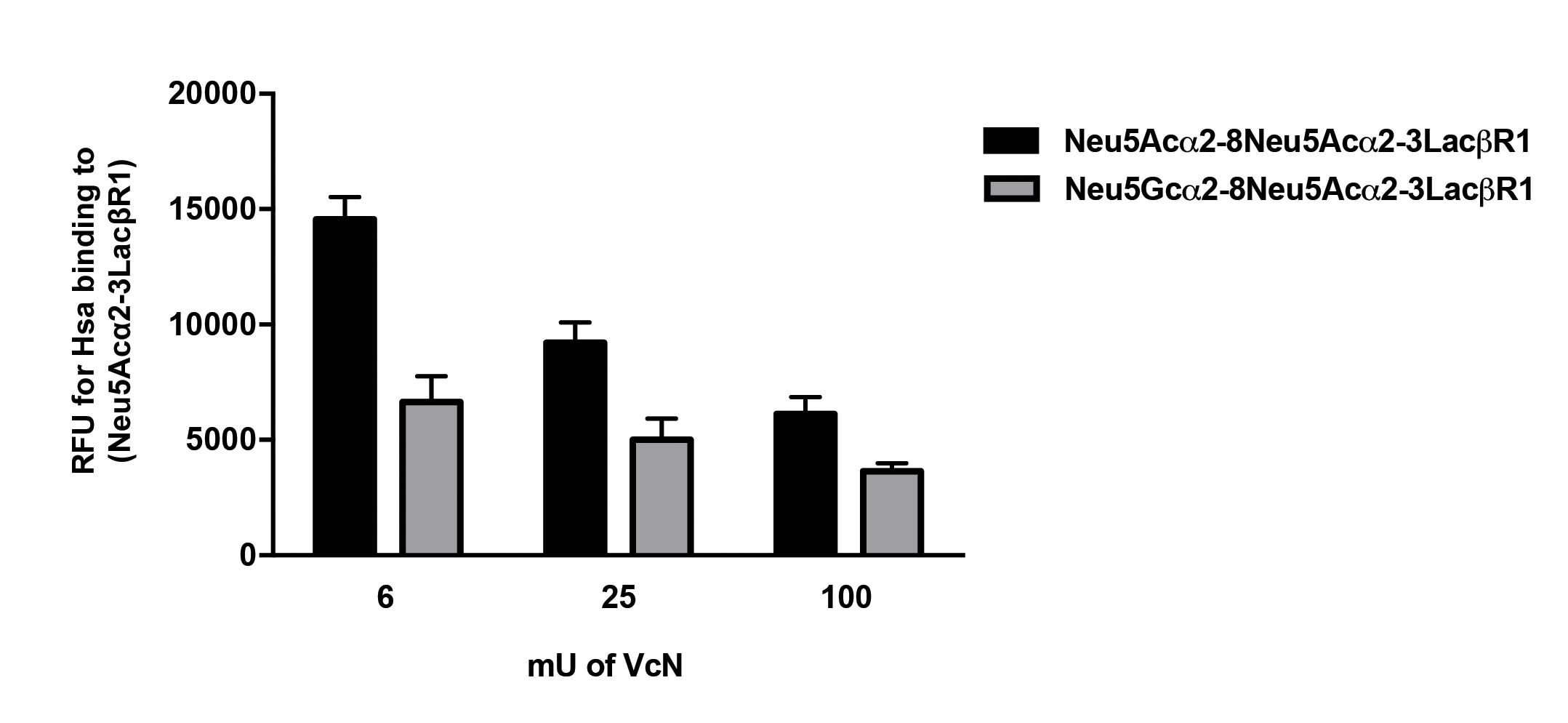

Supplement: S1 Fig — Glass slides printed with the indicated synthetic structures were incubated with high amounts of VcN. The resulting structures were probed with bacterial Has adhesin to evaluate the cleavage of α2-3-linked mono sialic acid. (TIF) [file ppat.1007133.s001.tif]

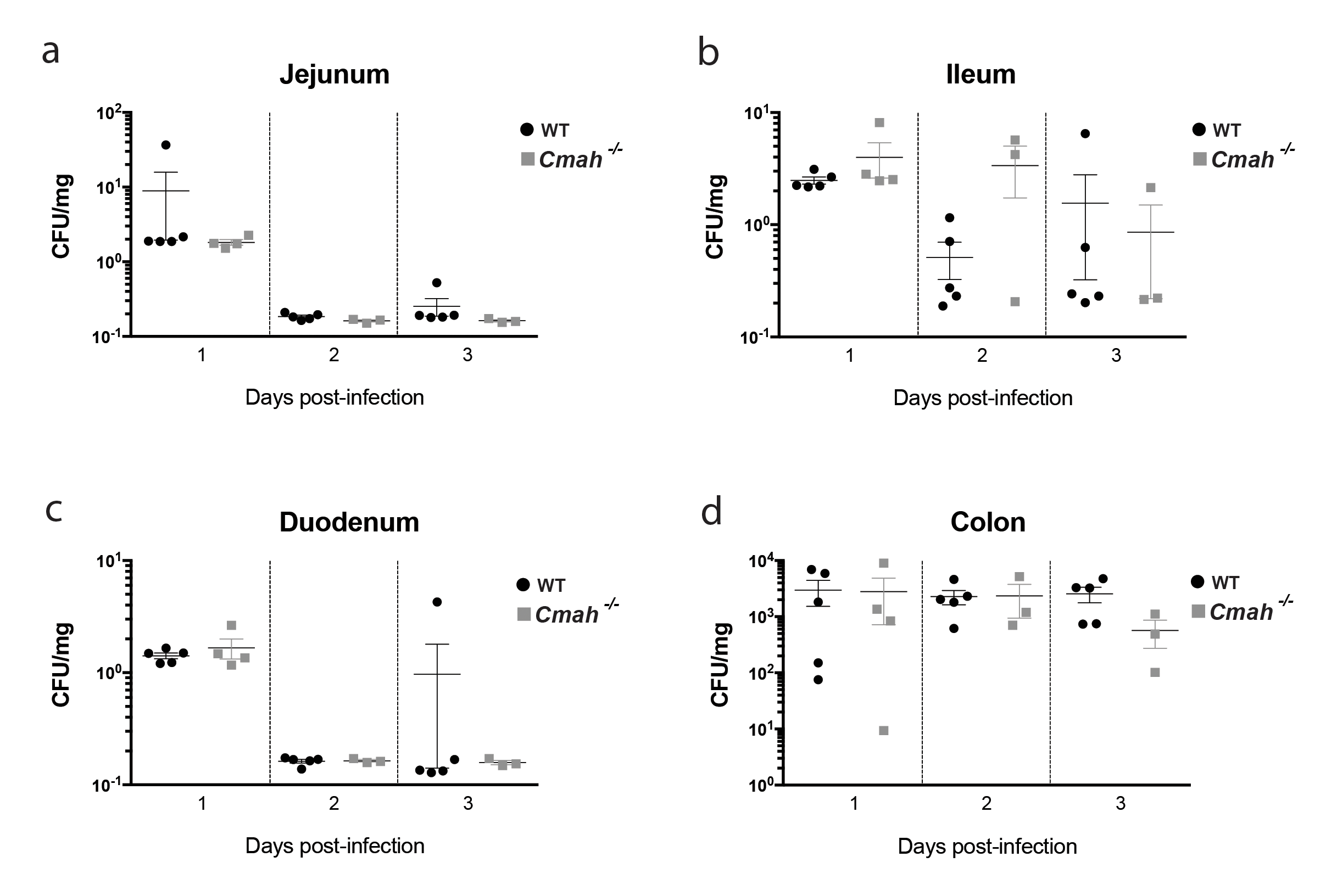

Supplement: S2 Fig — WT and Cmah-/- mice were treated with 5mg/mL of streptomycin for 24 h prior to oral infection and with 0.25mg/mL of streptomycin during the remaining experiment time, both in the drinking water. The animals were infected with 2x10^8^ CFU’s of V. cholerae and small intestine fractions divided into jejunum (a), ileum (b) duodenum (c), and the colon (d) were homogenized for Cholera CFU counts after serial dilutions were plated on (TCBS) agar media. CFU recovery was determined in tissue fragments harvested from days 1, 2 and 3 after infection. (TIF) [file ppat.1007133.s002.tif]

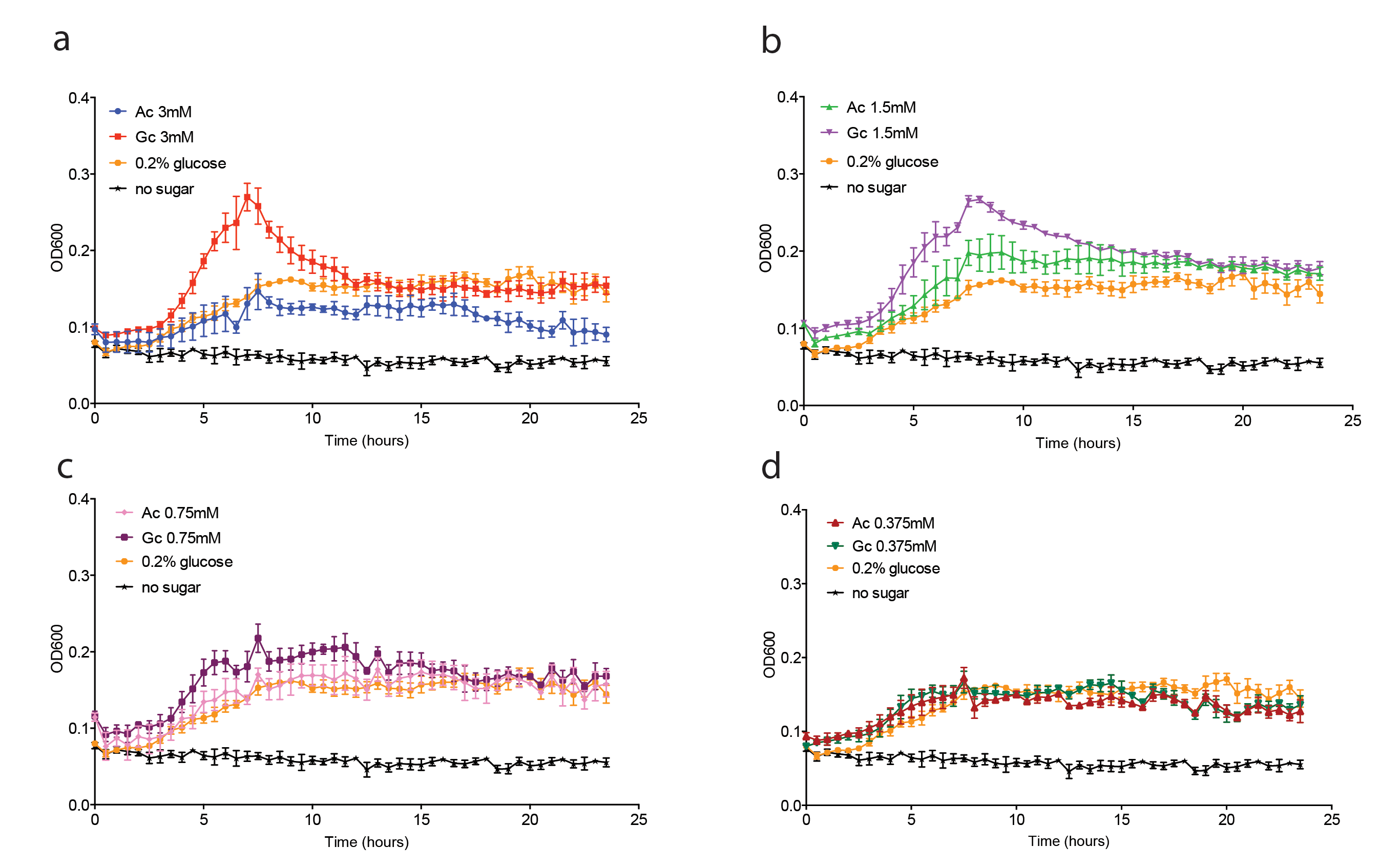

Supplement: S3 Fig — Bacterial growth in minimal M9 medium supplemented with 0.2% of glucose and concentrations of 3mM (a) 1.5 mM (b) 0.75mM (c) and 0.0375mM (d) of Neu5Ac or Neu5Gc was measured at each 30 min interval during 24h using Bioscreen C system. Data is representative of three independent experiments, with each condition done in quadruplicate. (TIF) [file ppat.1007133.s003.tif]
